# Examination of basic motor skills in children and adolescents

**DOI:** 10.3389/fphys.2023.1346750

**Published:** 2024-02-28

**Authors:** Soner Karadeniz, Ceren Suveren, Yasin Arslan, Tebessüm Ayyıldız Durhan, Tülay Ceylan, Faruk Albay, Hamza Küçük, Levent Ceylan

**Affiliations:** ^1^ Fatih Sultan Mehmet Secondary School, Sivas, Turkiye; ^2^ Coaching Education, Faculty of Sports Sciences, Gazi University, Ankara, Turkiye; ^3^ Yasar Doğu Faculty of Sport Sciences, Ondokuz Mayıs University, Samsun, Turkiye; ^4^ Faculty of Sports Sciences, Sivas Cumhuriyet University, Sivas, Turkiye

**Keywords:** KTK, gross motor coordination (GMC), balance, obesity, motor coordination (MC)

## Abstract

**Aim of the study:** The aim of this study was the investigation of basic motor skills in 5–14-year-old boys and girls.

**Materials and Methods:** A total of 842 primary school children, 421 boys and 421 girls, participated in the study. 13.3% of the participants were 5–6 years old, 29.5% were 7–8 years old, 21.5% were 9–10 years old, 16.4% were 11–12, and 19.4% were 13–14 years old. The balance skills of the participants were measured with the (Körperkoordinationstest für Kinder) KTK test.

**Findings:** When the classification of children according to KTK defining classes is examined, 40.7% are very good. When the children’s KTK Backward Balancing scores were examined, a statistically significant difference was found between gender and age groups (*p* < 0.05). Girls between the ages of 5–6 and 7–8 years had a higher score for KTK backward balance. KTK Total scores were examined according to the Body Mass Index groups, when the total scores of KTK were compared, the lowest scores were in the obese group.

**Conclusion:** According to the study results, age is an essential factor for balance skills. As the age increased, the overall scores of the KTK increased. It was determined that girls’ KTK backward balancing scores were higher than boys. According to BMI results, the balance performances of obese children were found to be lower than the other groups. This difference can be explained by the negative effect of obesity in this age group. According to these results, it may be recommended to observe and improve the balance performances of obese children.

## 1 Introduction

The development that begins in the womb continues to accelerate after birth. Movements that initially begin with reflex movements later form the basis for basic motor skills (Berk, 2015). In the period from birth to 2 years of age, children’s postural reflexes and primitive movements provide the opportunity to experience movement patterns and form the basis for the realization of voluntary movement skills in childhood (Gallahue, 1989).

In the early childhood years, primitive motor skills later expand to include basic movement skills such as walking, running, jumping and throwing, and become a motor activity with specific movement forms that form the basis for more advanced and specialized movement activities (Gabbard, 2021; Makaracı et al., 2022; Pamuk et al., 2023). Motor skills are the level of performance that individuals demonstrate in various motor actions that require one or more specific skills, including the coordination of fine and gross motor skills necessary for active participation in daily life (Sigmundsson, et al., 2021). The childhood motor skills assessment determines whether the child has movement skills appropriate to their developmental level and age. As a result of the assessment, identifying children with motor deficits and conducting studies to address these deficits is extremely important so that the child can properly master basic motor skills later in life (Gallahue, 1989).

Motor development, on the other hand, is a complex process and represents a sequential and continuous process of change that occurs in functional capacity and motor behavior throughout life, depending on age (Haywood et al., 2011; Haywood and Getchell, 2021; Makaracı et al., 2021). In particular, the biomechanics and function of the ankle joint, together with sensorimotor control and core stability, have a major influence on postural stability. The connections in the movement chains of the upper and lower extremities involved in strength, balance and movement control are maximized by the trunk muscles as they are located in the center of the body. Sensorimotor control creates a solid foundation for proper muscle recruitment and timing (De Oliveira et al., 2021; Şahin et al., 2022).

Since development in childhood forms the basis for motor development in the following years, it is extremely important to determine the level of motor development of children in preschool and school (Valentini et al., 2015). To this end, many countries have developed various test batteries (Wiart and Darrah, 2001; Barnett and Peters, 2004; Rudd et al., 2016; Bisi et al., 2017). These test batteries should allow us to reliably assess children’s motor development, and they should not only be easy to use but also be able to assess the developmental process of children at different ages (Valentini et al., 2015). Due to the importance of motor assessment for both diagnostic purposes and for determining intervention strategies, measurement instruments have been developed/validated over the years in different countries around the world and used in research to assess motor performance in children and adolescents (de Medeiros et al., 2016). The main purpose of studying children’s motor competence is to determine the level of motor development and the factors that influence motor development, to develop programs to improve motor development, and to determine the relationship of motor development to other domains (Bisi et al., 2017; Nascimento et al., 2019). In addition, superior balance is essential in many sports in order to reach the highest level of competition and avoid lower limb injuries. Balance is positively related to improved athletic performance and negatively related to lower limb sports injuries (Kiers et al., 2013). For this reason, it is important to determine the balance performance of children and adolescents. Determining the motor development, motor behaviour and motor performance of children and adolescents is essential to analyse the developmental processes of age groups. The results can be used to identify deficits in the motor development process of children and adolescents. In this way, training programmes can be created to improve motor performance. The aim of this study is to determine the motor performance of children and adolescents.

## 2 Material and methods

### 2.1 Participants

The research group consists of kindergarten and primary school children studying in 10 different schools. The sample of the study consists of students aged 5–14 years. A total of 842 (girls: *n* = 421; boys: *n* = 421) students took part in the study. The study complies with the ethical principles laid down in the Declaration of Helsinki. Kindergartens and elementary school were visited and the necessary permissions were obtained to conduct the study. Before each study conducted in the kindergartens and elementary school, the teachers received the necessary information about the purpose of the study and the studies were conducted under the supervision of the teachers. The schools where the measurements were to be taken were visited 1 day before, the area where the study was to be conducted was set up and then the test materials were arranged in the appropriate areas.

### 2.2 Anthropometric measurements

The height of the students who participated in the study was measured using a measuring device (SECA 804 Hamburg, Germany), with the students standing with bare feet and in an upright position. The length from crown to sole was measured by ensuring that the back of the head, back, hips and back of the feet were touching a flat wall and standing in an upright position (Lohman et al., 1988). Weight was measured using (SECA 213 Hamburg, Germany), ensuring that students were lightly clothed and barefoot (Lohman et al., 1988). The body mass index (weight/height^2^) (kg/m^2^) of all students was calculated from the height and weight measurements.

### 2.3 Gross motor coordination measurements

The KTK test, which was first developed by Kiphard and Schilling (1974) and modernized again in 2007, was used to measure the motor skills of the participants. The KTK test consists of four physical tests.

1. Backward balancing; The child walks backwards to 3 bars placed parallel to each other, 300 cm long, 5 cm high and gradually decreasing in width (6.0, 4.5, 3.0 cm), and makes 3 attempts for each bar.
2. Single foot bounce; The child is ready to start 1–2 steps ahead of the jumping station. He must jump to the other side by jumping on one leg and proceed by jumping at least two more times. Scoring is done by performing the same area with the other foot.
3. Jump to the sides; The child jumps from one side of a wooden slat with a length of 60 cm, a width of 4 cm and a height of 2 cm to the other side, with two feet, for 15 s, and each jump is worth a point.
4. Side stepping; In this test, the child stands on a platform with both feet, bends down, puts the other platform to the side using both hands, and passes over it. This movement is repeated for 20 s and 1 point is given for each platform it passes on.

KTK test results are evaluated according to the categories “insufficient motor coordination” (MK < 56), “severe motor disability” (MK 56–70), “moderate motor disability” (MK 71–85), “normal” (MK 86–115)”, “good” (MK 116–130) and “very good” (MK 131–145+) (Kiphard and Schilling, 1974; Kiphard and Schilling, 2007). Before starting the motor skill tests, the supervisor makes the necessary explanations for the test and then gives the student the right to try it once.

### 2.4 Statistical analysis

Normality assumptions and homogeneity tests were carried out before starting to analyze the data obtained. As a result of the Shapiro-Wilk and Levene tests performed, the data was found to have a normal distribution (*p* > 0.05). In the study, frequency (n), percentage (%), mean and standard deviation are given as values of descriptive statistics. The *t*-test was used to compare the genders and the one-way analysis of variance was used to compare the BMI groups. Duncan’s multiple comparison test was used to determine the difference between the groups. A significance level of 5% was considered statistically significant. The SPSS 21 program was used to analyze the data.

## 3 Results

Findings regarding the sociodemographic characteristics of the children who participated in the study are given in Table 1.

**TABLE 1:** Socio-demographic characteristics of the participants.

Gender	n	%
Girl	421	50
Boy	421	50
Age		
5–6	112	13.3
7–8	248	29.5
9–10	181	21.5
11–12	138	16.4
13–14	163	19.4
KTK Descriptive Classes		
Very Good	343	40.7
Good	119	14.1
Normal	226	26.8
Moderate Motor Impairment	83	9.9
Severe Motor Impairment	42	5.0
Insufficient Motor Coordination	29	3.4
Body Mass Index		
Under Weight (UW)	22	2.6
Normal Weight (NW)	473	56.2
Over Weight (OW)	130	15.4
Obese (OB)	217	25.8
**Total**	842	100.0

When examining the classification of children according to the KTK definition classes, 40.7% are very good, 14.1% good, 26.8% normal, 9.9% moderately motor impaired, 5.0% severe. It was found that 3.4% fell into the class of motor deficits and 3.4% into the class of insufficient motor coordination in Table 2.

**TABLE 2:** Comparison results for KTK sub-dimensions according to children’s gender, age groups and gender*age group interactions.

Variables	Age	Gender	Girl		Boy	Age	General
		n	Mean±sd	n	Mean±sd	n	Mean±sd
KTK Backward Balancing	5–6	67	28.30 ± 10,27^F^	45	23.73 ± 11,13^G^	112	26.46 ± 10.81^d^
	7–8	126	37.73 ± 12.85^D^	122	33.73 ± 11,38^E^	250	35.76 ± 12.28^c^
	9–10	86	45.17 ± 12.98^AB^	95	40.44 ± 14.96^CD^	248	42.69 ± 14.46 ^ab^
	11–12	64	41.75 ± 12.32^B−D^	74	40.80 ± 12.01^CD^	138	41.23 ± 12.12^b^
	13–14	78	43.07 ± 10.38^A−C^	85	46.10 ± 10.26^A^	163	44.65 ± 10.40^a^
	General	421	39.35 ± 13.15	421	37.91 ± 13.77	842	38.63 ± 13.48
KTK Single Leg Jump	5–6	67	9.94 ± 10,19^E^	45	9.95 ± 9.80^E^	112	9.94 ± 9.99^d^
	7–8	126	12.67 ± 9.90^DE^	122	13.19 ± 11,32^DE^	250	12.93 ± 10.60^c^
	9–10	86	14.33 ± 11,14^CD^	95	14.98 ± 12.40^CD^	248	14.67 ± 11.79 ^bc^
	11–12	64	14.87 ± 9.95^CD^	74	17.50 ± 11.36^BC^	138	16.28 ± 10.77^b^
	13–14	78	18.96 ± 16.96^B^	85	26.44 ± 11.02^A^	163	22.86 ± 14.62^a^
	General	421	14.07 ± 12.09	421	16.68 ± 12.51	842	15.38 ± 12.36
KTK Sideways Jump	5–6	67	28.74 ± 8.92	45	30.08 ± 11.76	112	29.28 ± 10.13^e^
	7–8	126	40.65 ± 11.57	122	39.30 ± 10.27	250	39.98 ± 10.95^d^
	9–10	86	49.75 ± 15.16	95	47.81 ± 11.81	248	48.73 ± 13.51^c^
	11–12	64	65.59 ± 11.65	74	62.66 ± 12.16	138	64.02 ± 11.98^b^
	13–14	78	66.33 ± 11.84	85	67.47 ± 13.39	163	66.92 ± 12.65^a^
	General	421	49.16 ± 18.14	421	50.03 ± 17.45	842	49.59 ± 17.79
Stepping Sideways on the KTK Platform	5–6	67	13.46 ± 2,75^DE^	45	13.00 ± 3,19^E^	112	13.27 ± 2.93^e^
	7–8	126	16.03 ± 3.27^CD^	122	15.54 ± 3,62^C−E^	250	15.79 ± 3.45^d^
	9–10	86	17.70 ± 4.15^C^	95	17.51 ± 4.01^C^	248	17.60 ± 4.07^c^
	11–12	64	22.56 ± 3.46^B^	74	23.06 ± 3.96^B^	138	22.83 ± 3.73^b^
	13–14	78	23.08 ± 4.15^B^	85	27.58 ± 22.54^A^	163	25.43 ± 16.64^a^
	General	421	18.26 ± 5.02	421	19.47 ± 11.76	842	18.86 ± 9.05
KTK Grand Total	5–6	67	80.45 ± 23.59^E^	45	76.78 ± 28.07^E^	112	78.97 ± 25.43^e^
	7–8	126	107.09 ± 27.03^D^	122	101.77 ± 26.93^D^	250	104.47 ± 27.06^d^
	9–10	86	126.98 ± 31.94^C^	95	120.72 ± 32.36^C^	248	123.69 ± 32.23^c^
	11–12	64	144.37 ± 28.73^B^	74	142.84 ± 31.29^B^	138	143.55 ± 30.03^b^
	13–14	78	150.26 ± 28.39^B^	85	163.53 ± 31.58^A^	163	157.18 ± 30.73^a^
	General	421	120.58 ± 36.78	421	123.06 ± 40.72	842	121.82 ± 38.80

Variables	Age	Gender	Gender*Age
KTK Backward Balancing	***	**	**
KTK Single Leg Jump	***	**	**
KTK Sideways Jump	***	NP	NP
Stepping Sideways on the KTK Platform	***	NP	*
KTK Grand Total	***	NP	*

NP: not important, *p < 0.05 **p < 0.01; ***p < 0.001 A,B,C: expresses the difference between lines, A,B: Expresses the difference between gender*age groups (interactions).

According to the children’s gender, age groups and gender-age group interactions, the interactions between KTK balancing backwards, KTK one-legged jumping, KTK walking sideways on the platform and KTK total score are statistically significant (*p* < 0.05). When examining the KTK sideways jump values, the interactions between gender and age groups are not statistically significant (*p* > 0.05).

When Table 3 was examined, comparing genders by different age groups, significant differences were found between genders in KTK backward balancing scores in age groups 5–6, 7–8, 9–10 (*p* < 0.05), while there were significant differences in age groups 11–12 and 13–14. No significant differences were found between the KTK backward balancing scores. In the 13–14 age group, significant differences were found between genders in KTK one-legged jump and total score (*p* < 0.05).

**TABLE 3:** Results of comparison of genders according to different age groups for KTK values.

Ages	KTK	n	Girl	n	Boy	t	*p*
			Mean ± sd		Mean ± sd		
5–6	Back Balancing	67	28.30 ± 10.28	45	23.73 ± 11.13	2.228	0.028
	One Foot Jump	67	9.94 ± 10.20	45	9.96 ± 9.80	−0.008	0.994
	Jumping Sideways	67	28.75 ± 8.92	45	30.08 ± 11.76	−0.686	0.494
	Stepping Sideways on the Platform	67	13.46 ± 2.75	45	13.00 ± 3.20	0.816	0.416
	Grand total	67	80.45 ± 23.59	45	76.78 ± 28.07	0.747	0.456
7–8	Back Balancing	126	37.73 ± 12.85	122	33.72 ± 11.38	2.592	0.010
	One Foot Jump	126	12.67 ± 9.90	122	13.20 ± 11.32	−0.387	0.699
	Jumping Sideways	126	40.65 ± 11.57	122	39.30 ± 10.27	0.968	0.334
	Stepping Sideways on the Platform	126	16.03 ± 3.27	122	15.54 ± 3.62	1.100	0.272
	Grand total	126	107.09 ± 27.03	122	101.77 ± 26.93	1.551	0.122
9–10	Back Balancing	86	45.17 ± 12.98	95	40.44 ± 14.96	2.261	0.025
	One Foot Jump	86	14.33 ± 11.14	95	14.98 ± 12.40	−0.371	0.711
	Jumping Sideways	86	49.75 ± 15.16	95	47.81 ± 11.81	0.967	0.335
	Stepping Sideways on the Platform	86	17.70 ± 4.15	95	17.51 ± 4.01	0.319	0.750
	Grand total	86	126.98 ± 32.36	95	120.72 ± 32.36	1.308	0.193
11–12	Back Balancing	64	41.75 ± 12.32	74	40.79 ± 12.01	0.459	0.647
	One Foot Jump	64	14.87 ± 9.94	74	17.50 ± 11.36	−1.433	0.154
	Jumping Sideways	64	65.59 ± 11.65	74	62.66 ± 12.16	1.439	0.152
	Stepping Sideways on the Platform	64	22.56 ± 3.46	74	23.06 ± 3.96	−0.791	0.430
	Grand total	64	144.37 ± 28.73	74	142.84 ± 31.29	0.299	0.766
13–14	Back Balancing	78	43.07 ± 10.38	85	46.10 ± 10.26	−1.871	0.063
	One Foot Jump	78	18.96 ± 16.96	85	26.44 ± 11.02	−3.366	0.001
	Jumping Sideways	78	66.33 ± 11.84	85	67.47 ± 13.39	−0.572	0.568
	Stepping Sideways on the Platform	78	23.08 ± 4.15	85	27.58 ± 22.54	−1.735	0.085
	Grand total	78	150.26 ± 28.39	85	163.53 ± 31.58	−2.812	0.006

When examining the KTK backward balancing, the KTK jump on one leg and the overall KTK result as a function of the body mass index groups, a statistically significant difference was found between the groups for the variables examined (*p* < 0.05). No statistically significant difference was found between the groups for the variables KTK sideways jump and KTK sideways step on the platform (*p* > 0.05) in Table 4.

**TABLE 4:** Comparison results of Body Mass Index groups in terms of KTK values.

KTK	Body Mass index	n	Average	S.Deviation	*p*
KTK Backward Balancing (WB)	Under Weight (UW)	22	44,77^a^	10.73	
	Normal Weight (NW)	473	39,89^b^	13.81	
	Over Weight (OW)	130	39,50^b^	0.001	12.82
	Obese (OB)	217	34,74^c^	12.57	
KTK Single Leg Jump (JS)	Under Weight (UW)	22	17,77^a^	13.55	
	Normal Weight (NW)	473	17,05^a^	12.73	
	Over Weight (OW)	130	15,10^ab^	0.001	11.66
	Obese (OB)	217	11,64^b^	11.01	
KTK Sideways Jump (MS)	Under Weight (UW)	22	44.31^a^	10.70	
	Normal Weight (NW)	473	49.38 ^ab^	17.88	
	Over Weight (OW)	130	52.03^a^	0.204	17.96
	Obese (OB)	217	49.13 ^ab^	17.99	
Stepping Sideways on KTK	Under Weight (UW)	22	17.36	3.63	
Platform (HH)	Normal Weight (NW)	473	18.84	6.03	
	Over Weight (OW)	130	19.13	5.13	
	Obese (OB)	217	18.91	14.92	
KTK Grand Total	Under Weight (UW)	22	124.23	31.05	
	Normal Weight (NW)	473	124.62	38.76	
	Over Weight (OW)	130	125.66	38.04	
	Obese (OB)	217	113.17	38.95	

n, number; SD, standard deviation; WB, walking backward; JS, jumping sideways; MS, moving sideways; HH, hopping for height; UW, underweight; NW, normal weight; OW, overweight; OB, obesity.

When the BMIs of the children who took part in the study were compared by gender in different age groups, no statistically significant difference was found for any of the age groups included in the study (*p* > 0.05) in Table 5.

**TABLE 5:** Comparison results of BMIs in terms of gender according to different age groups.

Age	n	Girl	n	Boy	t	*p*
5–6	67	16.49 ± 3.24	45	17.37 ± 2.34	−1.561	0.121
7–8	126	17.41 ± 3.32	122	17.77 ± 3.49	−0.831	0.407
9–10	86	17.92 ± 3.82	95	18.40 ± 4.20	−0.787	0.433
11–12	64	18.52 ± 2.83	74	18.89 ± 3.92	−0.624	0.533
13–14	78	20.18 ± 3.78	85	19.34 ± 3.20	1.534	0.127

## 4 Discussion and conclusion

Motor competence is the ability to perform a movement completely and accurately as a result of experience and learning (Gallahue, 1989). Balance is critical in the development of children’s motor skills. Good balance allows children to succeed more in daily activities such as walking, running and jumping. A good balance performance affects children’s ability to synchronize their hands, feet, and trunk. For this reason, it is essential to investigate balance performance in children The assessment of children’s motor competence is essential for the early detection of motor performance problems, the determination of motor development levels and factors influencing motor development, and the development of programs to improve motor skills (Bisi et al., 2017; Nascimento et al., 2019). There are many motor test batteries for assessing children’s motor competence that have been developed for a specific age group and goal (Wiart and Darrah, 2001; Barnett and Peters, 2004; Rudd et al., 2015; Bisi et al., 2017). By obtaining reliable results with these test batteries used, the aim is to accurately assess motor skill development data and diagnose motor deficits at an early stage (de Souza et al., 2007; Carminato, 2010; Medina-papst and marques, 2010). In this study, the Körperkoordinations test Für Kinder (KTK) developed by Kiphard and Schilling (1974) and modernized again in 2007 was used. When examining the children’s KTK backward balancing scores, a statistically significant difference was found between the genders and age groups (*p* < 0.05). It was found that the KTK backward balancing scores of girls were higher than those of boys. In general, KTK backward balancing scores were higher in 13–14-year-old children than in 11–12-, 9–10-, 7-8- and 5-6-year-old children, and the lowest score was found in 5-6-year-old children (*p* < 0.05). According to Berk (2015), although girls are more gifted than boys in motor skills that require balance and attention in infancy, they are less gifted than boys in motor skills such as catching, throwing and running. The reason for this is that girls and boys want to engage in different physical activities from childhood onwards. While boys prefer sports that require performance, such as basketball and football, girls prefer skills such as jumping rope and sewing.

There are studies in the literature that find similar results to our research. Laukkanen, et al. (2017), conducted a study on 64 children between the ages of 5-7, and determined that girls got better scores in KTK backward balance and sideways stepping on the platform, and boys got better scores in sideways jumping and single-footed jumping. Giuriato et al. (2021), found that boys performed better than girls in the total scores of three of the four KTK subtests, while girls performed better than boys in the KTK backward balance skill. Coppens et al. (2021), determined in their study that boys got higher scores than girls in KTK sideways jumping, KTK sideways stepping on the platform and KTK one-legged jump in every age group, and that girl got higher scores than boys in KTK balance. There are studies in the literature that find similar results to our research. Laukkanen et al. (2017), conducted a study on 64 children between the ages of 5-7, and determined that girls got better scores in KTK backward balance and sideways stepping on the platform, and boys got better scores in sideways jumping and single-footed jumping. Giuriato et al. (2021), found that boys performed better than girls in the total scores of three of the four KTK subtests, while girls performed better than boys in the KTK backward balance skill. Coppens et al. (2021), determined in their study that boys got higher scores than girls in KTK sideways jumping, KTK sideways stepping on the platform and KTK one-legged jump in every age group, and those girls got higher scores than boys in KTK balance. In our research, it was determined that the scores obtained increased with increasing age. According to this result we found in our research, it can be thought that there is an increase in motor performance with increasing age. A gradual improvement in motor performance occurs with increasing age. This development in motor performance is seen as a general phenomenon in child development, depending on the person’s predispositions as well as the accumulation of motor experiences, including both motor learning and motor control development processes (Haywood and Getchell, 2021). Quantitative differences in motor skills tend to increase parallel to levels of experience, physical growth and development, and changes in neurological function. It has been determined that with advancing age, most individuals experience increases in characteristics such as body size, muscle mass, strength, cardiorespiratory capacity and perceptual-motor skills (Gabbard, 2021).

There are many studies that find an increase in children’s motor performance with age and confirm the role of age in motor performance. Godoi Filho et al. (2021) conducted a study with a total of 531 children aged 4–15 years and found that children’s scores on the KTK total score increased with age. Moreira et al. (2019) found in their study of a total of 565 Brazilian schoolchildren aged 5–10 years that older children performed significantly better than younger children. The results found in the study and the studies conducted support each other. Coppens et al. (2021) conducted a study with a total of 2,271 children aged 6–10 years and found that children in the older age group performed significantly better than children in the younger age group and that BC improved significantly with age.

According to the results of our study, it can be assumed that BMI has a direct influence on motor performance and has a negative effect on motor performance. Especially with the development of technology, children’s participation in physical activity decreases day by day (Dollman et al., 2005). Correspondingly, the number of children with obesity and low physical fitness is increasing significantly (Booth et al., 2012).

Given the key role that motor skills play in participation in health-promoting physical activities, early diagnosis of motor deficits or problems in overweight and obese children is therefore very important. These pre-pubertal children are at an appropriate age to address problems with their motor performance. Therefore, physical activities that support a healthy body weight from an early age can have a positive impact on children’s motor performance during this period and provide the foundation for good health that is maintained from pre-puberty into adulthood (Cools et al., 2009). Antunes et al. (2015), based on the results of their study of 1276 children aged 6–14 years, found that normal weight children performed significantly better on motor performance tests than their obese peers. Lopes et al. (2018), based on the results of his study of 3738 children aged 6–10 years, found that overweight/obese children showed lower motor performance than their normal and underweight peers. Marmeleira et al. (2017) found that normal weight children had better motor skills than overweight/obese children. In their study of 1961 children aged 6–13 years, Battaglia et al. (2021) found that overweight/obese children showed significantly poorer motor performance than their normal-weight peers in all age groups, with the exception of 6- to 7-year-old boys.

## 5 Conclusions

One of the results we found in our study shows that age has a significant impact on the development of basic movement skills. It was found that with increasing age, the scores for KTK backward balancing, KTK one-legged jumping, KTK sideways jumping, KTK walking sideways on the platform and KTK in general increased. It was found that the KTK of backward balancing was higher in girls than in boys. This result can also be explained by the effect of the developmental difference in age. It was found that the total score of 13–14 years old children was higher than that of 11–12, 9–10, 7–8 and 5–6 years old children, the lowest score was obtained by 5–6 years old children, and no significant difference was found between girls and boys in KTK total scores.

Obesity is thought to have a negative impact on the development of motor skills and motor performance in children, which can lead to a reduction in motor competence compared to children of normal weight. Based on this finding, it is assumed that it is important to diagnose motor disorders in obese children at an early stage. At the same time, obese children should be offered regular physical activities to improve their motor skills. Balance is crucial for participation in physical activities and interaction with peers during childhood. Physical activity strategies that promote a healthy body weight from an early age can have a positive impact on motor performance in preschool and elementary school-aged children and be maintained into adulthood, laying the foundation for good health. The study was limited to sedentary children. Further studies on groups of athletes are recommended.

## Data Availability

The datasets presented in this study can be found in online repositories. The names of the repository/repositories and accession number(s) can be found in the article/Supplementary material.
